# Supramolecular Solvent-Based Liquid Phase Microextraction Combined with Ion-Pairing Reversed-Phase HPLC for the Determination of Quats in Vegetable Samples

**DOI:** 10.3390/toxics7040060

**Published:** 2019-11-26

**Authors:** Sophon Hem, Netsirin Gissawong, Supalax Srijaranai, Suthasinee Boonchiangma

**Affiliations:** Materials Chemistry Research Center, Department of Chemistry and Center of Excellence for Innovation in Chemistry, Faculty of Science, Khon Kaen University, Khon Kaen 40002, Thailand; sophonhem@gmail.com (S.H.); Netsirin_Gissawong@hotmail.com (N.G.); supalax@kku.ac.th (S.S.)

**Keywords:** supramolecular solvent (SUPRAS), quaternary ammonium compounds (quats), liquid phase microextraction (LPME), ion-pairing reverse phase high performance liquid chromatography (IP-RPHPLC)

## Abstract

In this study, we used anion supramolecular solvent (SUPRAS) prepared from a mixture of an anionic surfactant, sodium dodecyl sulfate (SDS), and a cationic surfactant, tetrabutylammonium bromide (TBABr), as the extraction solvent in liquid phase microextraction (LPME) of paraquat (PQ) and diquat (DQ). The enriched PQ and DQ in the SUPRAS phase were simultaneously analyzed by ion-pairing reversed-phase high performance liquid chromatography. PQ and DQ were successfully extracted by LPME via electrostatic interaction between the positive charge of the quats and the negative charge of SUPRAS. PQ, DQ, and ethyl viologen (the internal standard) were separated within 15 min on a C18 column, with the mobile phase containing 1-dodecanesulfonic acid and triethylamine, via UV detection. The optimized conditions for the extraction of 10 mL aqueous solution are 50 μL of SUPRAS prepared from a mixture of SDS and TBABr at a mole ratio of 1:0.5, vortexed for 10 s at 1800 rpm, and centrifugation for 1 min at 3500 rpm. The obtained enrichment factors were 22 and 26 with limits of detection of 1.5 and 2.8 µg L^−1^ for DQ and PQ, respectively. The precision was good with relative standard deviations less than 3.86%. The proposed method was successfully applied for the determination of PQ and DQ in vegetable samples and recoveries were found in the range of 75.0% to 106.7%.

## 1. Introduction

A group of quaternary ammonium compounds, also known as quats, has been used as herbicides and anticholinergic drugs. Among quats, paraquat (PQ) or 1,1′-dimethyl-4,4′-bipyridinium dichloride, commercial name Gramoxone and diquat (DQ) or 1,1′-ethylene-2,2′-bipyridyldiylium dibromide, have been commonly used as non-selective herbicides [1,2]. They are fast-acting contact herbicides that inhibit photosynthesis, so they have been used as pre-harvest desiccants and defoliants and for industrial and aquatic weed control [3]. Quats are highly water soluble, can easily contaminate water, and adsorb in clay and soil, to be taken up by plants and subsequently enter the food chain [4,5]. The maximum residue limits of paraquat established by the European Commission (Regulation 396/2005) in vegetables is 0.02 mg/kg. The ingestion of the most toxic quats, PQs, into the human body can have long-term health effects, such as Parkinson’s disease, liver failure, heart failure, lung damage, and skin cancer [6]. Therefore, it has been banned in some countries [7,8].

Chromatographic techniques, such as gas chromatography (GC) and high performance liquid chromatography (HPLC), have been used as powerful techniques for simultaneous analysis of quats [9,10,11,12,13]. For GC analysis, derivatization is required to increase the volatility of quats [14]. Ion exchange chromatography (IEC) and its analog technique, capillary electrophoresis (CE), are suitable for analysis of ionic compounds like quats [15,16]. However, IEC columns are quite expensive and CE has some limitations due to low sensitivity. Ion-pairing reversed-phase HPLC (IP-RPHPLC) uses an inexpensive reversed-phase column with an aqueous mobile phase containing ion pairing agents and is used as an alternative technique for the determination of ionic compounds. Many reports have been published on the determination of quats using IP-RPHPLC with a mass spectrometer (MS), and alternatively UV detection can be used [12,13,17,18,19]. 

Sample preparation is an essential step in the analytical field. Nowadays, trends for sample preparation are focused on microextraction techniques. Solid phase extraction (SPE), especially ion exchange sorbents, has been extensively used for extraction of quats [3,19,20,21]. Many strategies, such as dispersive solid phase extraction (DSPE) [22,23] and headspace solid phase microextraction (HS-SPME) [24], have been used. Ion-pair SPE has been used as sample preparation for the determination of quaternary ammonium herbicides [12,13,25]. However, SPE has some drawbacks due to the many steps involved, i.e., conditioning, loading, and eluting. In addition, the sorbents of SPE are usually costly and their preparation procedures consume large amounts of solvents. Another mode of microextraction based on solvent extraction, liquid phase microextraction (LPME), has received considerable attention. The important features of LPME are the use of a small volume of extraction solvent, rapid process, and high enrichment factor [26,27,28,29]. Recently, LPME has been focused on green solvents, such as ionic liquid [30], to eliminate the use of toxic solvents. 

Supramolecular solvents (SUPRASs), water immiscible nanostructured liquids, produce colloid amphiphilic solutions through sequential self-assembly and coacervation [31,32]. They have the potential to be used as the extraction solvents for various compounds due to the tunability of solvent properties [33]. In addition to ease of preparation and low cost, SUPRASs are considered green solvents as they are non-volatile and non-flammable [34,35,36]. SUPRASs have been used as the extraction solvent in LPME for determination of various analytes in different samples, including benzimidazolic fungicides in environmental water [37], mecoprop and dichlorprop in soil [38], endocrine disruptors in sediment [39], Sudan dyes in foodstuffs [40], and tetracyclines in food samples [41]. 

In this study, we investigated LPME combined with HPLC equipped with a UV detector as a sensitive method for simultaneous analysis of quats. PQ and DQ (the structures shown in Table S1) were enriched by LPME using SUPRAS as the extraction solvent prior to their analysis by IP-RPHPLC. SUPRAS was prepared easily from a mixture of sodium dodecyl sulfate (SDS) and tetrabutylammonium bromide (TBABr). The proposed method was applied for the determination of the studied quats in vegetables. In addition, a LPME mechanism is proposed. To the best of our knowledge, this is the first time trace quats have been determined using SUPRAS-based LPME for enrichment of quats prior to their analysis by IP-RPHPLC with common UV detection. 

## 2. Materials and Methods 

### 2.1. Chemicals and Reagents

All chemicals and reagents were of at least analytical reagent grade. Quats standards, paraquat dichloride, and diquat dibromide hydrate were purchased from Dr. Ehrenstorfer GmbH (Augsburg, Germany). Ethyl viologen dibromide was obtained from Sigma-Aldrich (St. Louis, MO, USA). SDS and TBABr were purchased from Ajax Finechem (NSW, Australia). Acetonitrile (HPLC grade) was purchased from Merck (Darmstadt, Germany). 1-dodecanesulfonic acid sodium salt and triethylamine (TEA) were obtained from Fluka (Tokyo, Japan). Ortho-phosphoric acid (H_3_PO_4_) and hydrochloric acid were obtained from QRëC (Auckland, New Zealand). Aluminum chloride (AlCl_3_) was purchased from Sigma-Aldrich (St. Louis, MO, USA). Potassium bromide (KBr) was obtained from Ajax Finechem (NSW, Australia) and sodium hydroxide was purchased from Carlo Erba (Marseille, France).

All standard stock solutions were prepared in de-ionized water with a specific resistivity of 18.2 MΩ cm from RiO_s_™ Type I Simplicity 185 (Millipore, Burlington, MA, USA).

### 2.2. Instrumentation

The chromatographic separations were conducted on an Agilent 1220 LC VL system consisting of Agilent 1260 Infinity II photodiode array detector (DAD), a binary pump, and a 20 μL Rheodyne injection loop. OpenLAB CDS Chemstation software was used for data acquisition. The separation was performed on a Purospher^®^ STAR RP-18 end-capped (150 × 4.6 mm I.D., 5μm) from Merck (Darmstadt, Germany). 

A vortex mixer (50 Hz) from Scientific Industries, Inc. (Bohemia, NY, USA), and a centrifuge (H-11n, Kokusan, Tokyo, Japan) were used for extraction and centrifugation in the extraction step, respectively. An ultrasonic water bath (35 kHz and 320 W) with temperature control from Bandelin Sonorex (Berlin, Germany) was also used. A transmission electron microscope (TEM; TECNAI G2 20, FEI, Hillsboro, OR, USA) and zeta potential analyzer (Zetasizer Nano ZS, Malvern, U.K.) were used for the morphology of SUPRAS and charge on the surface of SUPRAS, respectively. 

### 2.3. SUPRAS Preparation 

An aliquot (1.00 mL) of 50 mmol L^−1^ SDS and 0.50 mL of 50 mmol L^−1^ TBABr were placed in a centrifuge tube and 1.5 g of AlCl_3_ was added. Then, the mixture solution was adjusted to 10 mL with de-ionized water. The solution was mixed using vortex for 20 s at the fixed speed of 1800 rpm, and a cloudy solution was observed. After, the solution was centrifuged at 3500 rpm for 5 min for phase separation. Finally, the SUPRAS phase (upper phase) was collected using a syringe and maintained in a glass vial at room temperature before use.

### 2.4. LPME Procedure

Standard or sample solution (10.00 mL) was placed in a 10 mL centrifuge tube and 50 µL of SUPRAS (from Section 2.3) was added. The solution was vortexed for 10 s at 1800 rpm, and a colloidal solution was observed. After centrifugation at 3500 rpm for 1 min, the supernatant was withdrawn. Finally, the residue was dissolved in 20 µL acetonitrile before injection to HPLC for quats analysis (Section 2.5).

### 2.5. Chromatographic Separation of Quats

In preliminary studies, reversed-phase HPLC mode was investigated using various organic modifier and buffered mobile phases, which resulted in the incomplete separation of quats. Thus, ion pairing reversed phase-HPLC (IP-RPHPLC) was used. As the studied quats possess positive charges, the mobile phase containing negatively charged ion pairing (1-dodecanesulfonic acid) was investigated. The optimum mobile phase was a mixture of acetonitrile and aqueous solution of 10 mM TEA containing 5 mM 1-dodecanesulfonic acid sodium salt and 0.1 M KBr adjusted to pH 3 with *o*-phosphoric acid under isocratic elution with the ratio of 30/70 (*v*/*v*). The flow rate was 0.8 mL min^−1^. Using the optimum mobile phase, the three studied quats (PQ, DQ, and ethyl viologen as the internal standard) were separated within 15 min. The maximum absorption wavelengths were selected for monitoring PQ and DQ at 254 and 310 nm, respectively.

### 2.6. Sample Preparation

Vegetable samples, including Chinese cabbage, radish, onion, and cabbage, were purchased from local markets in Khon Kaen, Thailand. The vegetable samples preparation were prepared following the procedure described previously [11] with some modifications. The samples were chopped and blended. Then, the homogenized sample (2.5 g) was mixed with 10 mL water and sonicated for 15 min and centrifuged for 5 min at 3500 rpm. After, the supernatant was filtered through Whatman filter paper no. 1 to remove the solid residues. Finally, the filtrate (1.00 mL) was transferred to a centrifuge tube containing 9.00 mL water and was extracted following the LPME procedure described in Section 2.4. 

### 2.7. Validation Study

Calibration curves were obtained using the internal standard method. The peak area ratios of the analytes (paraquat and diquat) and internal standard (ethyl viologen) were plotted against the concentration of the analytes. Five concentrations of each analyte were studied. The limit of detection (LOD) was evaluated using a signal to noise (S/N) ratio of 3:1 and limit of quantitation (LOQ) was S/N of 10:1. In this study, the matrix-matched calibration curve was used for quantitative determination to compensate for matrix effects. Five different concentrations of the PQ and DQ standards ranging from 20 to 500 µg L^−1^ and 400 µg L^−1^ ethyl viologen were added to the sample filtrate (10 mL) and prepared according to Section 2.4. The precision was expressed as the relative standard deviation (%RSD). 

## 3. Results and Discussion

### 3.1. Optimization of SUPRAS-Based Liquid Phase Microextraction

In this study, LPME was used as the preconcentration method for quats before their analysis by IP-RPHPLC. The SUPRAS prepared from a mixture of SDS and TBABr was used as the extraction solvent. To obtain the optimum conditions for extraction, a 10 mL aqueous solution containing 0.1 mg L^−1^ of each quat was used throughout. Parameters affecting the extraction efficiency were studied and optimized including surfactant composition, SUPRAS volume, salt addition, pH, vortex time, and centrifugation time. The extraction efficiency is expressed in term of percentage extraction recovery (% ER) as follows [42]:

$$\%\;\mathrm{ER}\;=\;\frac{C_{\mathrm{ex}}}{C_{o}}\;\times \;100$$

where C_ex_ and C_o_ are the concentrations of analytes in the extraction phase and the initial concentration of analyte in the aqueous solution, respectively.

#### 3.1.1. Effect of Surfactant Composition (SDS:TBABr) 

**Figure 1:**
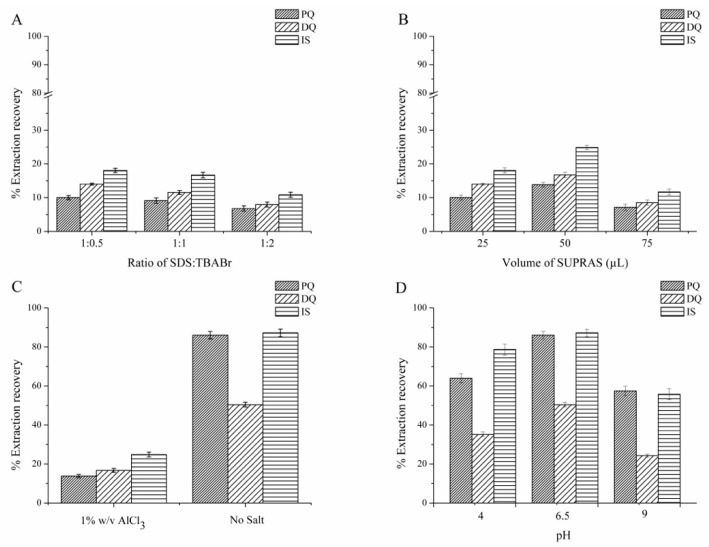
Effect of (**A**) anionic surfactant (SDS) to cationic surfactant (TBABr) mole ratio (SDS:TBABr), (**B**) supramolecular solvent (SUPRAS) volume, (**C**) salt addition, and (**D**) pH on the extraction efficiency. Extraction conditions: 10 mL standard solution (0.1 mg L^−1^ of each quat, pH 6.5), SDS:TBABr at mole ratio of 1:0.5, 50 µL of SUPRAS, no salt addition, vortex for 10 s at 1800 rpm, centrifugation for 1 min at 3500 rpm. HPLC conditions: Acetonitrile and 10 mM TEA containing 5 mM 1-dodecanesulfonic acid sodium salt and 0.1 M KBr adjusted to pH 3 with *o*-phosphoric acid under isocratic elution with the ratio of 30/70 (*v*/*v*), at a flow rate of 0.8 mL min^−1^.

Good extraction solvents should have a capability to effectively extract the target analytes and be compatible with the instruments being used [26]. The first parameter to be investigated for LPME was the type of extraction solvent. In this study, the SUPRAS was ex situ prepared from an anionic surfactant (SDS) and cationic surfactant (TBABr); therefore, the first parameter studied was the composition of these surfactants. We found that without the addition of salt, the phase separation between SUPRAS and the bulk aqueous was not obtained. The mole ratios of SDS and TBABr were investigated at 1:0.5, 1:1, and 1:2 of SDS:TBABr in the presence of 15% *w*/*v* AlCl_3_. The results in Figure 1A reveal that the highest quats extraction efficiency was obtained at the mole ratio 1:0.5 of SDS:TBABr. This may due to the highest negative charge on the surface of SUPRAS produced at this ratio, thus facilitating the interaction with the positive charge of the studied quats. Therefore, SUPRAS prepared in a 1:0.5 molar ratio of SDS:TBABr was used for further experiments.

#### 3.1.2. Effect of SUPRAS Volume

The volume of SUPRAS at a 1:0.5 ratio of SDS to TBABr was studied in the range of 25 to 75 µL. The results are shown in Figure 1B. The extraction efficiency increased with increasing SUPRAS volume from 25 to 50 µL, which dramatically decreased at 75 µL, potentially due to the dilution effect. Hence, 50 µL was chosen as the optimum SUPRAS volume for further studies. 

#### 3.1.3. Effect of Salt

Salts have been used as coacervating agents to induce phase separation between bulk the aqueous solution and micelle. The role of salt is to neutralize the charge of the micelle, as the formation of micelle from ionic amphiphiles is composed of two opposite forces, i.e., the hydrophobic attraction of non-polar tails and the repulsion of the ionic head group [31,42]. Salts affect the extraction efficiency via the salting out phenomenon, especially for ionic compounds [43]. 

In this study, 15% *w*/*v* AlCl_3_ was used as the coacervating agent in the SUPRAS preparation step. For the optimization of LPME on the type and volume of the extraction solvent (Section 3.1.1 and Section 3.1.2) 1% *w*/*v* AlCl_3_ was added. However, to further study the effect of salt on the extraction efficiency by LPME, we found that without the addition of salt, the extraction efficiency increased (Figure 1C). This may due to the salt competing or obstructing the migration of quats into the SUPRAS, thus decreasing the extraction efficiency [44]. Therefore, subsequent studies were performed without salt addition.

#### 3.1.4. Effect of pH

The effect of pH was studied with acidic (pH 4) and basic (pH 9) media by the adjustment with HCl and NaOH. Neutral pH (obtained without pH adjustment, pH 6.5) was also studied. The results in Figure 1D show that the highest extraction efficiency was obtained with neutral media and the extraction efficiency significantly decreased in an alkaline solution. This may be due to the instability of quats in an alkaline solution [26]. Therefore, pH 6.5 was selected as a suitable pH and used in subsequent experiments.

#### 3.1.5. Effect of Vortex Time 

In this study, the duration of vortex is defined as the extraction time. Vortex facilitates the mass transfer of the analytes to the extraction phase, thus increasing the extraction [45]. The vortex rotational speed was fixed at 1800 rpm and vortex time was investigated from 5 to 20 s. The results (Figure S1) reveal that the extraction efficiency was constant after 10 s. Thus, vortex time was fixed at 10 s.

#### 3.1.6. Effect of Centrifugation Time 

Centrifugation is a process to accelerate the separation of the extraction phase from the aqueous solution. The centrifugation time was studied from 1 to 5 min at 3500 rpm. The results (Figure S2) showed that centrifugation time has a minimal effect on the extraction efficiency. Thus, a centrifugation time of 1 min was chosen. 

### 3.2. Proposed Extraction Mechanism 

**Figure 2:**
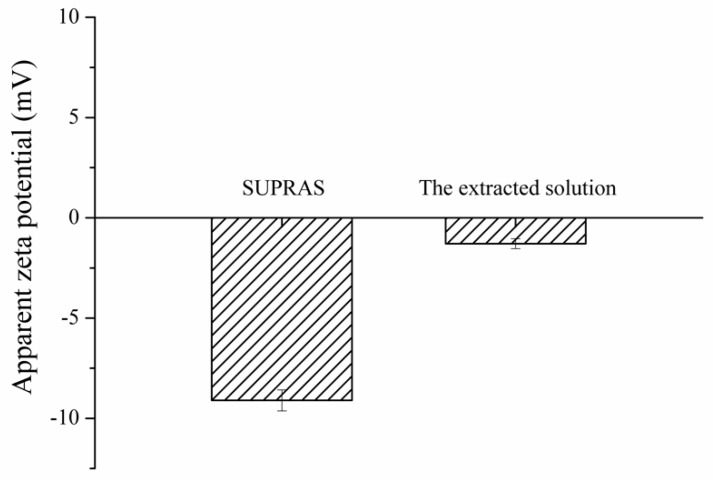
Zeta potential of SUPRAS and the extracted solution.

The proposed extraction mechanism is the electrostatic interaction between the positive charge of quats and the negative charge of SUPRAS. The results (Figure 2) show a zeta potential of SUPRAS solvent with negative potential (−9.10 mV) related to the ratio of anionic surfactant (SDS) to cationic surfactant (TBABr) of 1:0.5. After extraction, the extracted solution (SUPRAS phase containing quats) had a zeta potential of −1.29 mV. The charge potential of the extracted solution increased after the extraction of quats, confirming the electrostatic interaction mechanism.

**Figure 3:**
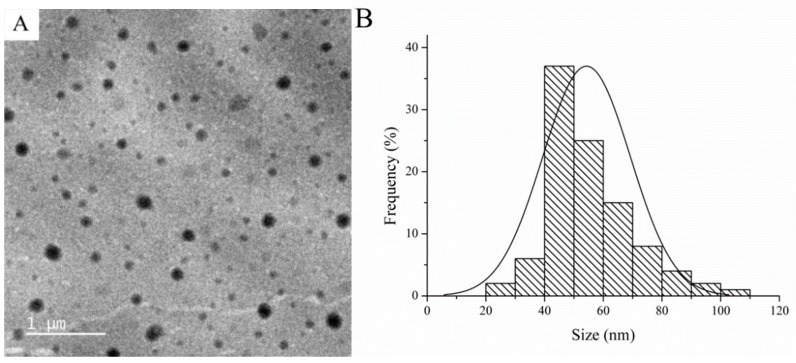
(**A**) Transmission electron microscopy (TEM) images and (**B**) particle sizes of SUPRAS.

The morphology and particle size of SUPRAS was investigated using TEM. TEM images revealed a spherical shape of SUPRAS (Figure 3A) with an average size of 50 nm (Figure 3B). 

### 3.3. Analytical Performance and Method Validation 

**Figure 4:**
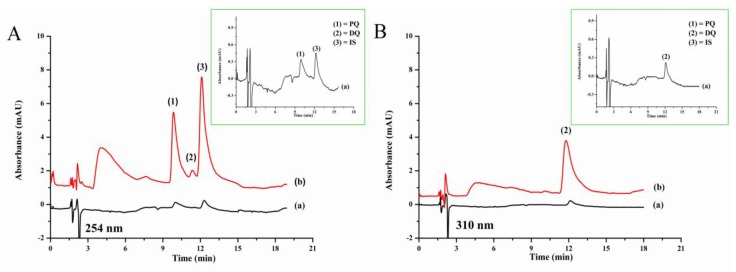
Chromatograms of standard quats (**A**) detected at 254 nm, (**B**) detected at 310 nm, and (a) without LPME (0.50 mg L^−1^ each) and (b) with LPME (0.10 mg L^−1^ each): 1, paraquat; 2, diquat; 3, ethyl viologen (IS).

Figure 4A shows the chromatogram of quats (0.5 mg L^−1^ each with direct IP-RPHPLC analysis and 0.1 mg L^−1^ each from LPME-IP-RPHPLC method) at 254 nm for the determination of paraquat and ethyl viologen. Figure 4B shows the detection at 310 nm for the determination of diquat. 

The analytical performance and validation of the proposed method (LPME-IP-RPHPLC) were determined using the optimum conditions. The results (Table 1) were compared with the direct analysis by IP-RPHPLC. The SUPRAS-based LPME method is effective for determining the enrichment of quats. Good linearity in the range of 7 to 250 µg L^−1^ with correlation coefficient higher than 0.998 were obtained. Limits of detection (LODs) and limits of quantitation (LOQs) were evaluated based on signal to noise ratios (S/N) of 3 and 10, providing LODs of 1.5 and 2.8 µg L^−1^, and LOQs of 5.6 and 8.0 µg L^−1^ for diquat and paraquat, respectively. The repeatability (intra-day) precision (*n* = 3) and reproducibility (inter-day) precision (*n* = 3 × 3) of the proposed method are expressed as the relative standard deviation (RSDs), with values less than 2.26% and 3.86%, respectively. The enrichment factor (EF) was evaluated using the ratio of the slopes of the linear equations both with and without SUPRAS-based LPME. The EFs of PQ and DQ were 22 and 26, respectively.

### 3.4. Comparison with Other Methods

The proposed method (LPME combined with IP-RPHPLC) was compared with the other methods (Table 2). The proposed method provides comparable sensitivity (LOD) to other methods using UV detection. With SUPRAS used as the extraction solvent, the proposed method is easy to prepare, cheap, and environmentally friendly. The proposed LPME is also rapid (only 1 min for the extraction).

### 3.5. Analysis of Samples

The proposed method was applied to various vegetable samples including Chinese cabbage, radish, onion, and cabbage. In this study, the matrix-matched calibration curve was used for quantitative determination to compensate for matrix effects. 

**Figure 5:**
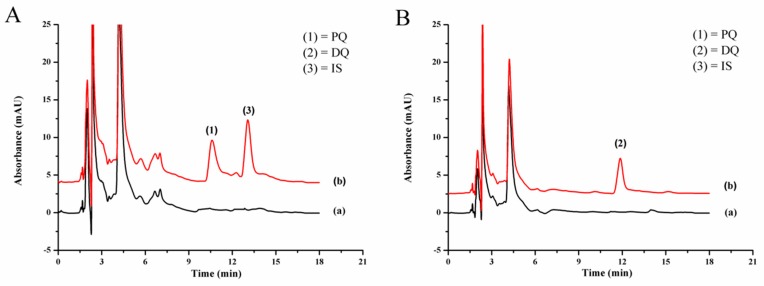
Chromatograms of onion sample (a) blank and (b) quats-spiked onion (0.4 mg kg^−1^ each) detected at (**A**) 254 nm and (**B**) 310 nm.

The studied quats were not found in any of the studied vegetable samples. Figure 5 shows the typical chromatograms of onion sample, showing no interference peaks. Figures S3–S5 show the chromatograms of the other samples. The accuracy of the proposed method was studied by spiking the standard paraquat and diquat at three concentration levels into the vegetable samples before analysis by LPME-IP-RPHPLC. The results are reported as the percentage recovery as summarized in Table 3. The recoveries obtained in the samples were in the range of 75.0% to 106.7%, which are acceptable [46]. 

## 4. Conclusions

This paper outlined a novel LPME method combined with IP-RPHPLC for the sensitive determination of quats (PQ and DQ). The schematic diagram of the proposed method is illustrated in Figure S6. SUPRAS was easily prepared from a mixture of anionic surfactant (SDS) and cationic surfactant (TBABr) at room temperature under phase separation induced by AlCl_3_. After LPME, the enriched quats in the extracted phase (SUPRAS) were determined by IP-RPHPLC equipped with a common UV detector. The proposed LPME showed an enrichment factor of 22 and 26 for paraquat and diquat, respectively. The proposed LPME only requires a short extraction time and could be used as an alternative extraction method for quat determination.

## Figures and Tables

**Table 1:** Analytical performance of the proposed method for the determination of the studied quats.

Analyte	Linear Range (µg L^−1^)	Linear Equation	*R^2^*	LOD (µg L^−1^)	LOQ (µg L^−1^)	EF
Diquat	7–250 (100–5000) ^a^	*y* = 0.0998*x* + 0.0633 (*y* = 0.0039*x* − 0.0986) ^a^	0.9996 (0.9990) ^a^	1.5 (25) ^a^	5.6 (80) ^a^	26
Paraquat	10–250 (100–5000) ^a^	*y* = 0.1031*x* − 0.7353 (*y* = 0.0047*x* − 0.5519) ^a^	0.9986 (0.9976) ^a^	2.8 (40) ^a^	8.0 (100) ^a^	22

^a^ Value of standard without SUPRAS-based liquid phase microextraction.

**Table 2:** The comparison of the proposed method with the other methods for the determination of quats.

Analyte	Sample	Sample Preparation	Extraction Solvent Volume (μL)	Extraction Time (min)	LOD (μg L^−1^)	Analytical Technique	Ref.
Paraquat	Blood	LLE (Chloroform- ethanol (7:3))	500	3	10	HPLC–DAD (258 nm)	[47]
Paraquat, diquat	Water	ISFME (ILs)	9.4	10	0.15–0.16	HILIC–DAD (256, 310 nm)	[29]
Paraquat	Vegetable	SPE (weak cation exchanger)	1000	NR	0.94 µg kg^−1^	HILIC–MS/MS	[11]
Paraquat	Water, Vegetable	DSPE (silica gel)	-	30	50	Spectro photometry	[25]
Paraquat	Water, Soil, and Vegetable	SALLE-RP- DLLME	2550	2.5	20	HPLC–UV (257 nm)	[28]
Paraquat, diquat	Water, Vegetable	SPE (alkyl-silica and resin SPE cartridge)	5000	NR	0.1–0.2	LC–MS	[23]
Paraquat, diquat	Vegetable	LPME (SUPRAS)	50	1	1.5–2.8	HPLC–DAD (254, 310 nm)	This work

Note: NR, not reported; ISFME, in situ solvent formation microextraction; IL, ionic liquid; HILIC, hydrophilic interaction liquid chromatography; DSPE, dispersive solid phase extraction; SALLE-RP-DLLME, salt-assisted liquid–liquid extraction coupled with reversed phase dispersive liquid–liquid microextraction.

**Table 3:** Recovery obtained from the determination of quats in the studied vegetable samples.

Analyte	Spiked (mg kg^−1^)	Chinese Cabbage (*n* = 3)			Radish (*n* = 3)			Onion (*n* = 3)			Cabbage (*n* = 3)		
		Found (mg kg^−1^)	Recovery (%)	RSD (%)	Found (mg kg^−1^)	Recovery (%)	RSD (%)	Found (mg kg^−1^)	Recovery (%)	RSD (%)	Found (mg kg^−1^)	Recovery (%)	RSD (%)
**Diquat**	0	ND	-	-	ND	-	-	ND	-	-	ND	-	-
	0.12	0.11	91.7	2.0	0.11	91.7	4.8	0.12	99.3	4.8	0.11	91.7	2.5
	0.20	0.16	80.0	5.5	0.18	90.0	3.0	0.17	82.7	1.7	0.19	95.0	3.8
	0.40	0.36	90.0	2.2	0.39	97.5	3.8	0.41	103.9	5.2	0.38	95.0	1.6
Paraquat	0	ND	-	-	ND	-	-	ND	-	-	ND	-	-
	0.12	0.10	83.3	1.5	0.10	83.3	7.9	0.13	106.7	4.0	0.12	100.0	3.5
	0.20	0.17	85.0	2.5	0.17	85.0	5.8	0.17	85.9	2.5	0.21	105.0	2.9
	0.40	0.30	75.0	3.8	0.36	90.0	4.1	0.35	86.5	1.1	0.39	97.5	1.6

Note: ND, not detected

## Supplementary Materials

The following are available online at https://www.mdpi.com/2305-6304/7/4/60/s1, Table S1: The structures and some chemical properties of paraquat and diquat, Figure S1: Effect of vortex time on the extraction efficiency, Figure S2: Effect of centrifugation time on the extraction efficiency, Figure S3: Chromatogram of cabbage, Figure S4: Chromatogram of Chinese cabbage, Figure S5: Chromatogram of radish, Figure S6: Schematic diagram of the proposed method.
