# Supplementary material for: Supramolecular Solvent-Based Liquid Phase Microextraction Combined with Ion-Pairing Reversed-Phase HPLC for the Determination of Quats in Vegetable Samples
Source: Toxics. 2019 Nov 26;7(4):60. doi: 10.3390/toxics7040060 (PMC6958504; doi:10.3390/toxics7040060)
Supplement: Supplementary file 1 [file toxics-07-00060-s001.pdf]

Supporting information

Supramolecular Solvent-Based Liquid Phase Microextraction Combined with Ion-Pairing Reversed-Phase HPLC for the Determination of Quats in Vegetable Samples

Sophon Hem [1], Netsirin Gissawong [1], Supalax Srijaranai [1] and Suthasinee Boonchiangma [1,*]

[1] Materials Chemistry Research Center, Department of Chemistry and Center of Excellence for Innovation in Chemistry, Faculty of Science, Khon Kaen University 40002, Thailand

* Correspondence: suthbo@kku.ac.th; Tel.: +66-4320-2222 ext.12243

***Corresponding author:**
Tel.: +66 43 202222 to 41 ext. 12243; fax: +66 43 202373
E-mail address: suthbo@kku.ac.th (S. Boonchiangma)

Table S1. The structures and some chemical properties of paraquat and diquat

Name	Structure	Molecular weight (g mol^{-1})	Solubility in water (g L^{-1})	Log K_{ow}
Paraquat dichloride (PQ)	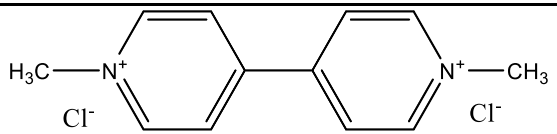	257	700	-4.70
Diquat dibromide (DQ)	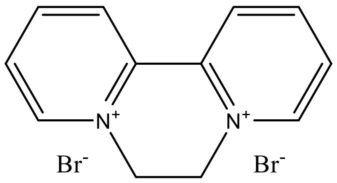	344	700	-4.60

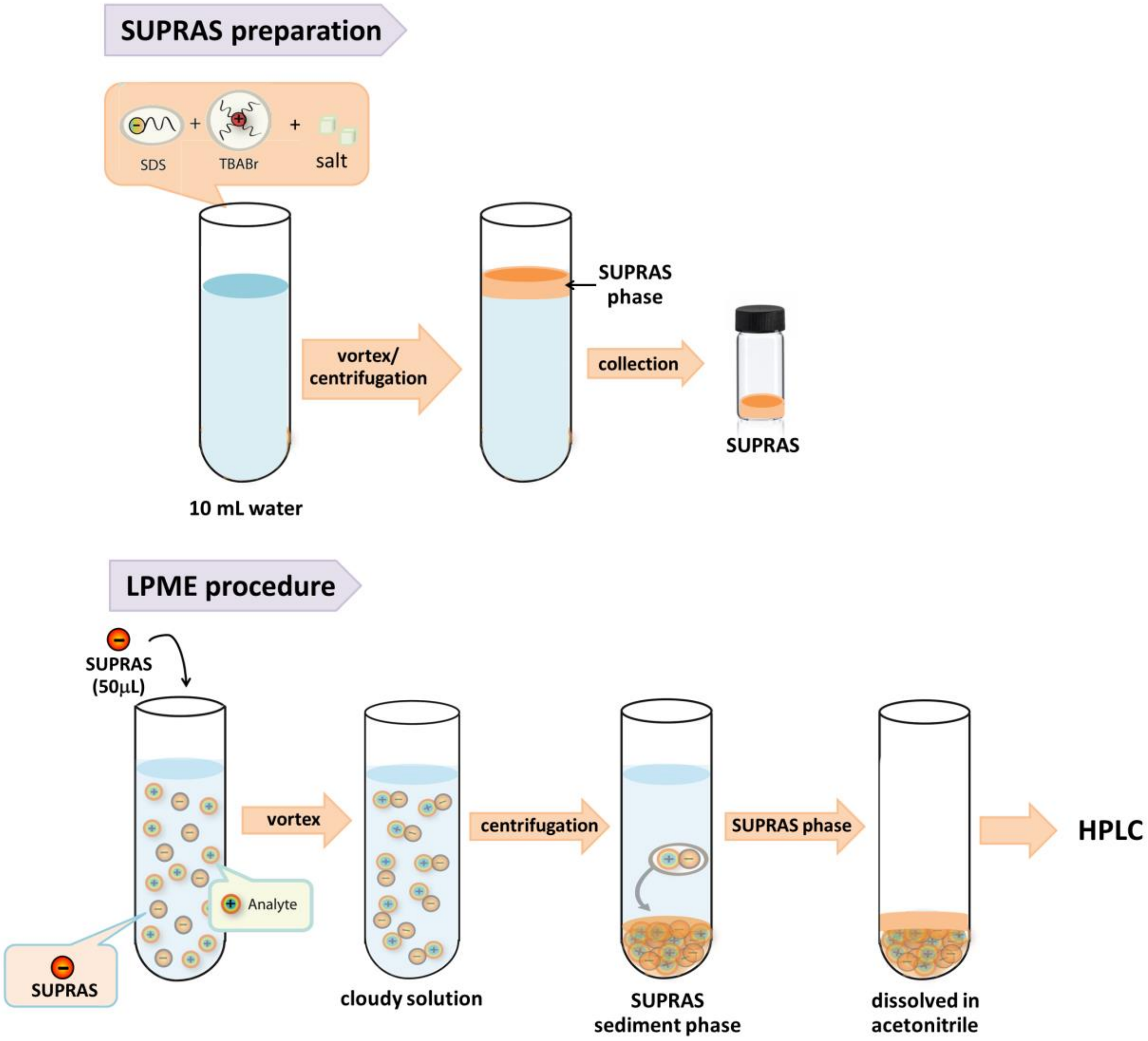

Figure S1 Schematic diagram of SUPRAS preparation and LPME procedure

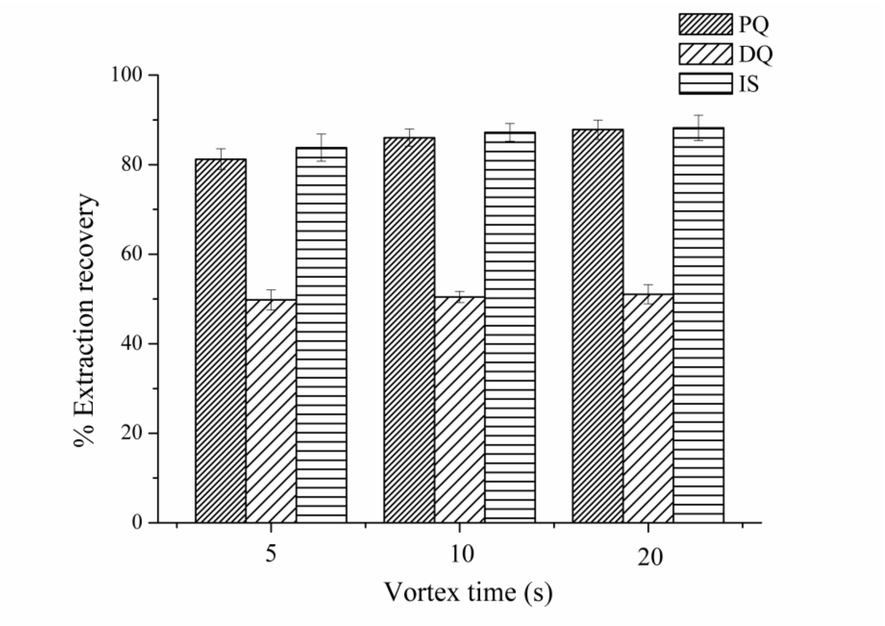

Figure S2 Effect of vortex time on the extraction efficiency

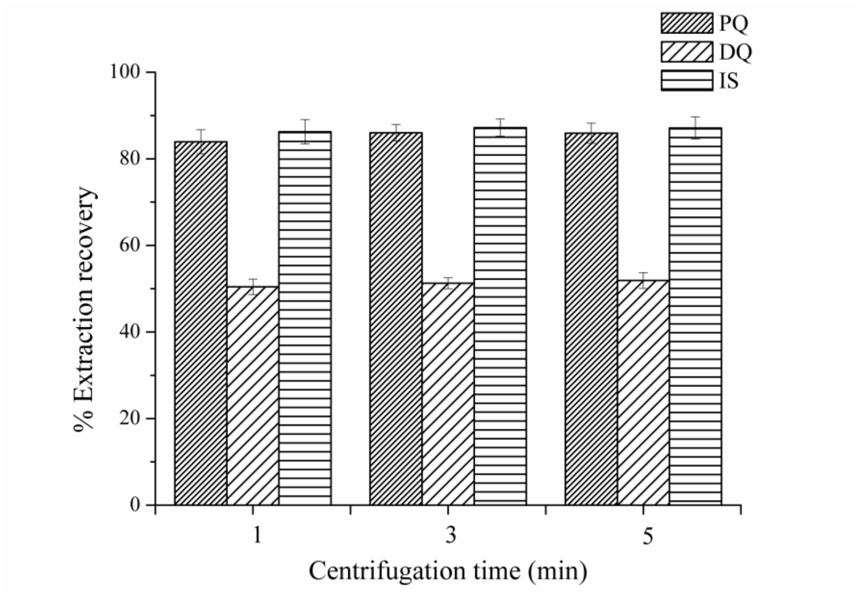

Figure S3 Effect of centrifugation time on the extraction efficiency

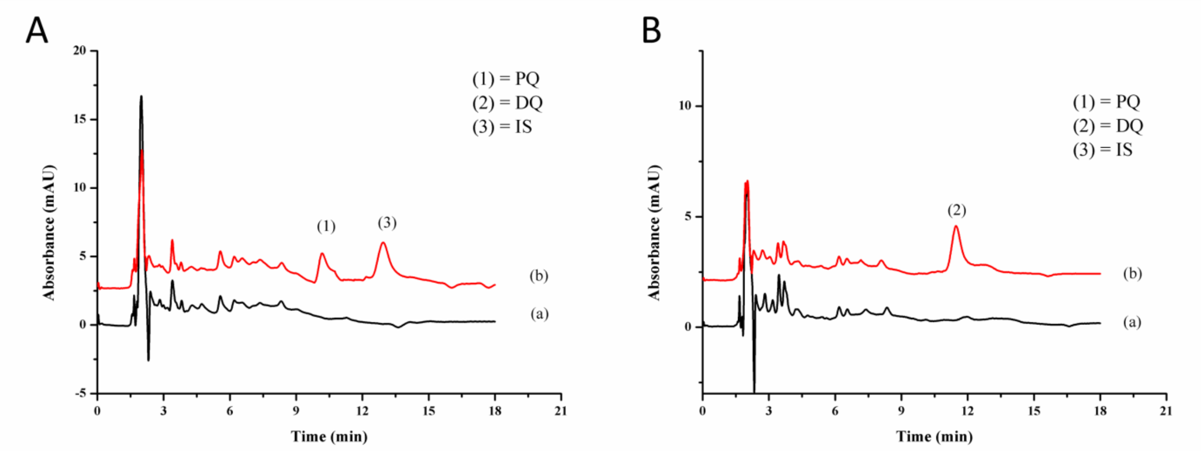

Figure S4 Chromatograms of cabbage sample (a) blank and (b) sample spiked quats (0.4 mg kg^{-1} each) using the optimum conditions of LPME, (A) detected at 254 nm (B) detected at 310 nm

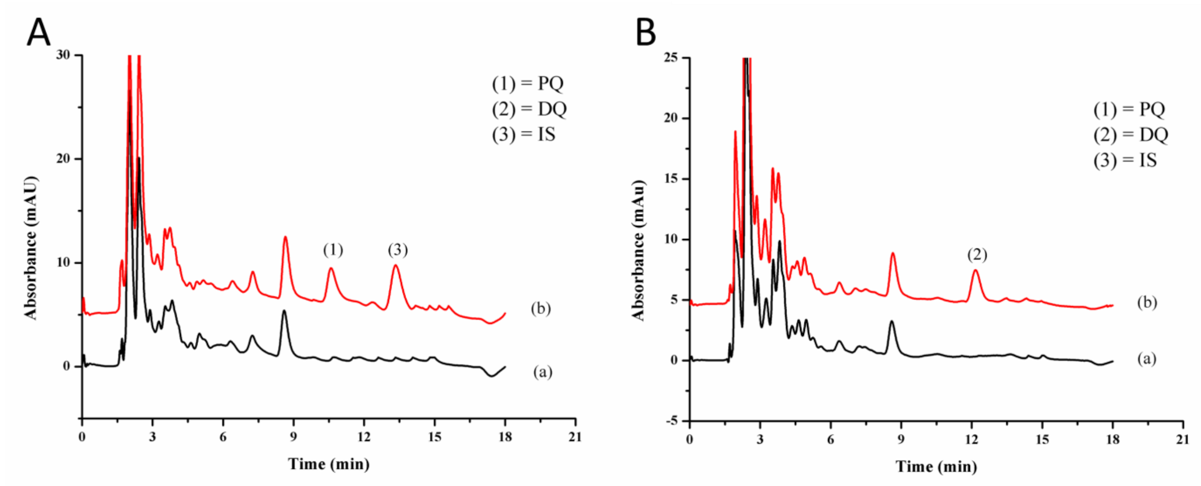

Figure S5 Chromatograms of Chinese cabbage sample (a) blank and (b) sample spiked quats (0.4 mg kg^{-1} each) using the optimum conditions of LPME, (A) detected at 254 nm (B) detected at 310 nm

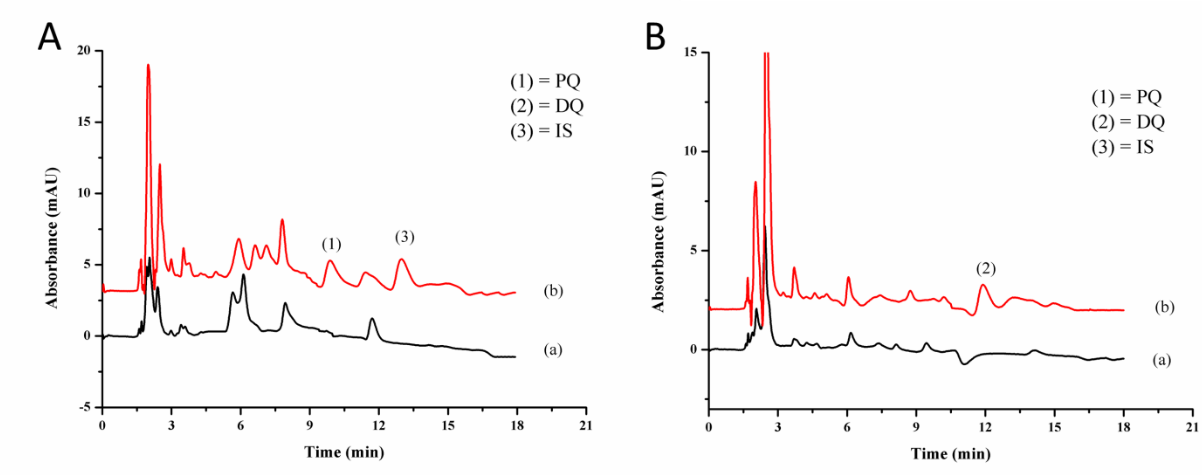

Figure S6 Chromatograms of Radish sample (a) blank and (b) sample spiked quats (0.4 mg kg^{-1} each) using the optimum conditions of LPME, (A) detected at 254 nm (B) detected at 310 nm
